# Characterization of NDM-Encoding Plasmids From *Enterobacteriaceae* Recovered From Czech Hospitals

**DOI:** 10.3389/fmicb.2018.01549

**Published:** 2018-07-10

**Authors:** Veronika Paskova, Matej Medvecky, Anna Skalova, Katerina Chudejova, Ibrahim Bitar, Vladislav Jakubu, Tamara Bergerova, Helena Zemlickova, Costas C. Papagiannitsis, Jaroslav Hrabak

**Affiliations:** ^1^Department of Microbiology, Faculty of Medicine and University Hospital in Plzen, Charles University, Plzen, Czechia; ^2^Faculty of Medicine, Biomedical Center, Charles University, Plzen, Czechia; ^3^Veterinary Research Institute, Brno, Czechia; ^4^Faculty of Science, National Centre for Biomolecular Research, Masaryk University, Brno, Czechia; ^5^National Reference Laboratory for Antibiotics, National Institute of Public Health, Prague, Czechia; ^6^Department of Clinical Microbiology, University Hospital and Faculty of Medicine in Hradec Kralove, Charles University, Hradec Kralove, Czechia

**Keywords:** NDM, metallo-β-lactamases, *Enterobacter xiangfangensis*, ST182, IncX3

## Abstract

The aim of the present study was to characterize sporadic cases and an outbreak of NDM-like-producing *Enterobacteriaceae* recovered from hospital settings, in Czechia. During 2016, 18 *Entrobacteriaceae* isolates including 10 *Enterobacter cloacae* complex (9 *E. xiangfangensis* and 1 *E. asburiae*), 4 *Escherichia coli*, 1 *Kluyvera intermedia*, 1 *Klebsiella pneumoniae*, 1 *Klebsiella oxytoca*, and 1 *Raoultella ornithinolytica* that produced NDM-like carbapenemases were isolated from 15 patients. Three of the patients were colonized or infected by two different NDM-like producers. Moreover, an NDM-4-producing isolate of *E. cloacae* complex, isolated in 2012, was studied for comparative purposes. All isolates of *E. cloacae* complex, except the *E. asburiae*, recovered from the same hospital, were assigned to ST182. Additionally, two *E. coli* belonged to ST167, while the remaining isolates were not clonally related. Thirteen isolates carried *bla*_NDM−4_, while six isolates carried *bla*_NDM−1_ (*n* = 3) or *bla*_NDM−5_ (*n* = 3). Almost all isolates carried *bla*_NDM_-like-carrying plasmids being positive for the IncX3 allele, except ST58 *E. coli* and ST14 *K. pneumoniae* isolates producing NDM-1. Analysis of plasmid sequences revealed that all IncX3 *bla*_NDM_-like-carrying plasmids exhibited a high similarity to each other and to previously described plasmids, like pNDM-QD28, reported from worldwide. However, NDM-4-encoding plasmids differed from other IncX3 plasmids by the insertion of a Tn*3*-like transposon. On the other hand, the ST58 *E. coli* and ST14 *K. pneumoniae* isolates carried two novel NDM-1-encoding plasmids, pKpn-35963cz, and pEsco-36073cz. Plasmid pKpn-35963cz that was an IncFIB(K) molecule contained an acquired sequence, encoding NDM-1 metallo-β-lactamase (MβL), which exhibited high similarity to the mosaic region of pS-3002cz from an ST11 *K. pneumoniae* from Czechia. Finally, pEsco-36073cz was a multireplicon A/C_2_+R NDM-1-encoding plasmid. Similar to other type 1 A/C_2_ plasmids, the *bla*_NDM−1_ gene was located within the ARI-A resistance island. These findings underlined that IncX3 plasmids have played a major role in the dissemination of *bla*_NDM_-like genes in Czech hospitals. In combination with further evolvement of NDM-like-encoding MDR plasmids through reshuffling, NDM-like producers pose an important public threat.

## Introduction

Acquired carbapenem-hydrolyzing β-lactamases are resistance determinants of increasing clinical importance in Gram-negative pathogens. Of these, NDM-1 metallo-β-lactamase (MβL) was first described in *Klebsiella pneumoniae* and *Escherichia coli* isolated in Sweden in 2008 from an Indian patient transferred from a New Delhi hospital (Yong et al., 2009). Since then, NDM-1-producing bacteria, including clinical isolates of *Enterobacteriaceae* and *Acinetobacter baumannii*, have been reported from the Indian subcontinent but also worldwide (Nordmann et al., 2011).

In Czechia, the occurrence of NDM-producing bacteria was rare, with only three sporadic cases being detected during 2011–2013. These cases included an NDM-1-producing *A. baumanni* isolated from a patient repatriated from Egypt (Hrabák et al., 2012), an NDM-4-producing strain of *Enterobacter cloacae* complex from a patient previously hospitalized in Sri Lanka (Papagiannitsis et al., 2013b) and a ST11 *K. pneumoniae* isolate carrying two NDM-1-encoding plasmids, from Slovakia (Studentova et al., 2015). However, an increase in the isolation frequency of NDM-like-producing *Enterobacteriaceae* from Czech hospitals was observed, during 2016.

Thus, the aim of the present study was to characterize the NDM-like producers detected in Czech hospitals, during 2016. Also, we describe the complete nucleotide sequences of representative *bla*_NDM_-like-carrying plasmids harbored by the studied isolates.

## Materials and methods

### Bacterial isolates and confirmation of carbapenemase production

In 2016, Czech hospitals referred a total of 410 *Enterobacteriaceae* isolates with a meropenem MIC of >0.125 μg/ml (EUCAST, 2012) to the National Reference Laboratory for Antibiotics. Species identification was confirmed by matrix-assisted laser desorption ionization-time of flight mass spectrometry (MALDI-TOF MS) using MALDI Biotyper software (Bruker Daltonics, Bremen, Germany). All isolates were tested for carbapenemase production by the MALDI-TOF MS meropenem hydrolysis assay (Rotova et al., 2017). Isolates that were positive by the MALDI-TOF MS meropenem hydrolysis assay were subjected to metallo-β-lactamase, KPC, and OXA-48 detection using the double-disc synergy test with EDTA, the phenylboronic acid disc test, and the temocillin disc test (Lee et al., 2003; Doi et al., 2008; Glupczynski et al., 2012), respectively. Additionally, carbapenemase genes (*bla*_KPC_, *bla*_VIM_, *bla*_IMP_, *bla*_NDM_, and *bla*_OXA−48_-like) were detected by PCR amplification (Poirel et al., 2004; Ellington et al., 2007; Naas et al., 2008; Yong et al., 2009). PCR products were sequenced as described below. Isolates positive for *bla*_NDM_-like genes were further studied. Moreover, the NDM-4-producing isolate of *E. cloacae* complex, recovered at the University Hospital Pilsen (Pilsen, Czechia) during 2012 (Papagiannitsis et al., 2013b), was included in this study for comparative purposes.

### Susceptibility testing

The MICs of piperacillin, piperacillin-tazobactam, cefotaxime, ceftazidime, cefepime, aztreonam, meropenem, ertapenem, gentamicin, amikacin, chloramphenicol, tetracycline, trimethoprim-sulfamethoxazole, ciprofloxacin, colistin, and tigecycline were determined by the broth dilution method (EUCAST, 2003). Data were interpreted according to the criteria of the European Committee on Antimicrobial Susceptibility Testing (EUCAST; www.eucast.org).

### Typing

All isolates were typed by multilocus sequence typing (MLST) (Diancourt et al., 2005; Wirth et al., 2006; Miyoshi-Akiyama et al., 2013; Herzog et al., 2014). The databases at https://pubmlst.org/ecloacae/, http://mlst.warwick.ac.uk/mlst/dbs/Ecoli, http://bigsdb.web.pasteur.fr/klebsiella and https://pubmlst.org/koxytoca/ were used to assign STs.

### Detection of β-lactamases

The β-lactamase content of all *bla*_NDM_-like-positive isolates was determined by isoelectric focusing (IEF). Bacterial extracts were obtained by sonication of bacterial cells suspended in 1% glycine buffer and clarified by centrifugation. Sonicated cell extracts were analyzed by IEF in polyacrylamide gels containing ampholytes (pH 3.5–9.5; AP Biotech, Piscataway, NJ). The separated β-lactamases were visualized by covering the gel with the chromogenic cephalosporin nitrocefin (0.2 mg/ml; Oxoid Ltd., Basingstoke, United Kingdom; Papagiannitsis et al., 2015).

On the basis of the IEF data, PCR detection of various *bla* genes was performed by the use of primers specific for *bla*_TEM−1_, *bla*_OXA−1_, *bla*_SHV_, *bla*_CTX−M_, and *bla*_CMY_, as reported previously (Pałucha et al., 1999; Pérez-Pérez and Hanson, 2002; Woodford et al., 2006; Coque et al., 2008). Both strands of the PCR products were sequenced using an ABI 377 sequencer (Applied Biosystems, Foster City, CA).

### Transfer of *bla*_NDM_-like genes

Conjugal transfer of *bla*_NDM_-like genes from the clinical strains was carried out in mixed broth cultures (Vatopoulos et al., 1990), using the rifampin-resistant *E. coli* A15 laboratory strain as a recipient. Transconjugants were selected on MacConkey agar plates supplemented with rifampin (150 mg/l) and ampicillin (50 mg/l). Plasmid DNA from clinical isolates, which failed to transfer *bla*_NDM_-like by conjugation, was extracted using a Qiagen Maxi kit (Qiagen, Hilden, Germany) and used to transform *E. coli* DH5α cells. The preparation and transformation of competent *E. coli* cells were done using calcium chloride (Cohen et al., 1972). Transformants were selected on Luria-Bertani agar plates with ampicillin (50 mg/l). Transconjugants or transformants were confirmed to be NDM-like producers by PCR (Yong et al., 2009) and the MALDI-TOF MS meropenem hydrolysis assay (Rotova et al., 2017).

### Plasmid analysis

To define the genetic units of the *bla*_NDM_-like genes, the plasmid contents of all NDM-producing clinical and recombinant strains were analyzed by pulsed-field gel electrophoresis (PFGE) of total DNA digested with S1 nuclease (Promega, Madison, WI, USA; Barton et al., 1995). Following PFGE, the DNA was transferred to a BrightStar-Plus positively charged nylon membrane (Applied Biosystems, Foster City, CA) and hybridized with digoxigenin-labeled *bla*_NDM_-like probe.

Plasmid incompatibility (Inc) groups were determined by the PCR-based replicon typing (PBRT) method (Carattoli et al., 2005; Johnson et al., 2012), using total DNA from transconjugants or transformants. Additionally, the IncR replicon was detected as described previously (García-Fernández et al., 2009).

### Detection of characteristic regions

Based on the results from Illumina sequencing (see below), six PCRs targeting characteristic regions of NDM-4-encoding IncX3 plasmids and genomes of ST182 isolates of *E. cloacae* complex sequenced during this study were designed. The selected regions included: (i) a Tn*3*-like transposon found in NDM-4-encoding IncX3 plasmids, and (ii) four insertions identified in the genome of Encl-922 (see section Comparative Analysis of *Enterobacter* Isolates). All NDM-producing clinical or recombinant strains were screened for the presence of the regions described above by the use of specific primers (see Table S1).

### Plasmid and chromosome sequencing

Ten plasmids were selected for complete sequencing. These plasmids were selected as representatives of different origins, plasmid sizes and hospitals. Additionally, clinical isolates Encl-922 and Encl-44578 were also selected for whole genome sequencing. The selected isolates were isolated 4-year apart (2012 and 2016).

Plasmid DNAs from transconjugants or transformants were extracted using a Qiagen Large-Construct kit (Qiagen, Hilden, Germany). Additionally, the genomic DNAs of clinical Encl-922 and Encl-44578 isolates were extracted using a DNA-Sorb-B kit (Sacace Biotechnologies S.r.l., Como, Italy). Multiplexed DNA libraries were prepared, using the Nextera XT Library Preparation kit, and 300-bp paired-end sequencing was performed on the Illumina MiSeq platform (Illumina Inc., San Diego, CA, USA) using the MiSeq v3 600-cycle Reagent kit. Initial paired-end reads were quality trimmed using the Trimmomatic tool v0.33 (Bolger et al., 2014) with the sliding window size of 4 bp, required average base quality ≥17 and minimum read length of 48 bases. Genomic DNA reads of clinical isolates of *E. cloacae* complex were consequently assembled using the de Bruijn graph-based *de novo* assembler SPAdes v3.9.1 (Bankevich et al., 2012), using k-mer sizes 21, 33, 55, 77, 99, and 127. For assembly of the plasmids, reads were mapped to the reference *E. coli* K-12 substrain MG 1655 genome (GenBank accession no. U00096) using the BWA-MEM algorithm (Li, 2013), in order to filter out the chromosomal DNA. Then, all the unmapped reads were assembled in the same way as described above. The sequence gaps were filled by a PCR-based strategy and Sanger sequencing. For sequence analysis and annotation, the BLAST algorithm (www.ncbi.nlm.nih.gov/BLAST), the ISfinder database (www-is.biotoul.fr/), and the open reading frame (ORF) finder tool (www.bioinformatics.org/sms/) were utilized. Comparative genome alignments were performed using the Mauve v2.3.1 program (Darling et al., 2010).

Antibiotic resistance genes were identified using the ResFinder 2.1 tool (https://cge.cbs.dtu.dk/services/ResFinder/) with an identity threshold of >90% (Zankari et al., 2012).

### Comparative analysis of clinical isolates of *E. cloacae* complex

Comparative genomic analysis of clinical isolates of *E. cloacae* complex was based on statistics calculated by QUAST v4.5 (Gurevich et al., 2013) and VarScan v2.3.9 (Koboldt et al., 2012) tools. All quality trimmed Illumina reads of Encl-922 were mapped to contigs of Encl-44578, employing BWA-MEM algorithm v0.7.12 (Li, 2013) and SAMtools v1.3 (Li et al., 2009), for the format conversions and analysis of the results. Then, single nucleotide polymorphisms (SNPs) and indels were detected employing VarScan with parameters set as follows: minimum read depth at a position = 6, minimum base quality at a position = 20 and minimum variant allele frequency threshold of 0.45. Moreover, SNPs and indels located in a region within 127 bp from any edge of a contig, as well as SNPs and indels harbored by contigs smaller than 2 kb were excluded from further analysis. Remaining SNPs and indels were also manually checked and refined by visualization of mapped data via Tablet v1.14.04.10 (Milne et al., 2013). Differences in assembly of *E. cloacae* complex genomes were inspected using QUAST's Icarus viewer (Mikheenko et al., 2016). In order to examine whether SNPs and indels were located in intergenic or coding regions, as well as to find out what are the differences in genetic information between studied isolates, contigs of clinical strains were annotated using Prokka v1.10 (Seemann, 2014). Genes harboring SNPs were compared against NCBI's conserved domain database (Marchler-Bauer et al., 2017) via CD-Search (Marchler-Bauer and Bryant, 2004) to identify conserved domain hits. Finally, sequencing data of clinical strains were examined for the presence of prophage sequences using PHAST web server (Zhou et al., 2011).

### Nucleotide sequence accession numbers

The nucleotide sequences of the pEsco-5256cz, pEncl-922cz, pRor-30818cz, pKpn-35963cz, pEsco-36073cz, pEncl-44578cz, pEnas-80654cz, pEnin-51781cz, pEsco-4382cz, and pKlox-45574cz plasmids have been deposited in GenBank under accession numbers MG252891, MG252892, MG252893, MG252894, MG252895, MG833402, MG833403, MG833404, MG833405, and MG833406, respectively. Whole genome assemblies of isolates of *E. cloacae* complex were deposited in NCBI under accession number PRJNA432167.

## Results

### Carbapenemase-producing *Enterobacteriaceae*

A total of 40 *Enterobacteriaceae* isolates showing carbapenemase activity on MALDI-TOF MS meropenem hydrolysis assay were recovered from Czech hospitals during 2016. PCR screening showed that 18 of the isolates were positive for *bla*_NDM_, 14 isolates were positive for *bla*_OXA−48_, while the remaining 8 isolates were positive for *bla*_KPC_.

### NDM-like-producing isolates

Altogether, 18 nonrepetitive isolates producing NDM-like carbapenemases were isolated from 15 patients in 2016. Among them, 10 were presumptively identified as belonging to *E. cloacae* complex, 4 were identified to be *E. coli*, while the remaining isolates belonged to unique species (*Enterobacter intermedius, K. pneumoniae, Klebsiella oxytoca*, and *Raoultella ornithinolytica*). A previous study showed that 16S rRNA gene sequence of *E. intermedius* was included within the cluster of the genus *Kluyvera*, and therefore, the transfer of *E. intermedius* to the genus *Kluyvera* as *Kluyvera intermedia* was proposed (Pavan et al., 2005). Three of the patients were colonized or infected by two different NDM-like producers (Table 1).

**Table 1:** Characteristics of NDM-like-producing *Enterobacteriaceae*.

**Isolate^a^**	**Isolation mn/yr (hospital)**	**Material (infection/colonization)**	**ST**	**β-Lactamase content**	**Size of NDM-encoding plasmid (kb)^b^**	**Replicon of NDM-encoding plasmid**	**Additional resistance markers**
***E. xiangfangensis***							
Encl-922	09/2012 (B1)	Rectal swab(colonization)	ST182	NDM-4, CTX-M-15, OXA-1, TEM-1	**~55 (53.683)**	IncX3	
Encl-66918	04/2016 (B1)	Rectal swab(colonization)	ST182	NDM-4, CTX-M-15, OXA-1, TEM-1	**~55**	IncX3	
Encl-89040	06/2016 (B1)	Bile(infection)	ST182	NDM-4, CTX-M-15, OXA-1, TEM-1	**~55**	IncX3	
Encl-44578	07/2016 (B1)	Venous catheter(infection)	ST182	NDM-4, CTX-M-15, OXA-1, TEM-1	**~55 (53.683)**	IncX3	
Encl-89485°	07/2016 (B1)	Bile(infection)	ST182	NDM-4, CTX-M-15, OXA-1, TEM-1	**~55**	IncX3	
Encl-91221	09/2016 (B1)	Throat swab(colonization)	ST182	NDM-4, CTX-M-15, OXA-1, TEM-1	**~55**	IncX3	
Encl-93141	10/2016 (B1)	Peritoneal catheter(infection)	ST182	NDM-4, CTX-M-15, OXA-1	**~55**	IncX3	
Encl-98042	11/2016 (B1)	Rectal swab(colonization)	ST182	NDM-4, CTX-M-15, OXA-1	**~55**	IncX3	
Encl-98047■	11/2016 (B1)	Rectal swab(colonization)	ST182	NDM-4, CTX-M-15, OXA-1, TEM-1	**~55**	IncX3	
Encl-98546	12/2016 (B1)	Rectal swab(colonization)	ST182	NDM-4, CTX-M-15, OXA-1, TEM-1	**~55**	IncX3	
***E. asburiae***							
Enas-80654°	07/2016 (B1)	Bile(infection)	NA	NDM-4, CTX-M-15	**~55 (53.683)**	IncX3	
***K. intermedia***							
Enin-51781	10/2016 (B1)	Rectal swab(colonization)	NA	NDM-4, CTX-M-15, OXA-1	**~55 (53.683)**	IncX3	
***E. coli***							
Esco-14290	06/2016 (B2)	Nasal swab(colonization)	ST167	NDM-5, CTX-M-15, TEM-1	*~45*	IncX3	
Esco-5256▴	07/2016 (B2)	Bronchoalveolar lavage(infection)	ST167	NDM-5, CTX-M-15, TEM-1	*~45 (46.161)*	IncX3	
Esco-36073	09/2016 (A1)	Urine(infection)	ST58	NDM-1, CMY-16, OXA-10, CTX-M-15, TEM-1	**~300 (300.958)**	IncR, IncA/C_2_	*floR, tet*(A), *strAB, sul2, aacA4, aphA7, dfrA14, arr-2, cmlA1, aadA1, aphA6, sul1*
Esco-4382■	12/2016 (B1)	Rectal swab(colonization)	ST69	NDM-4, CTX-M-15, TEM-1	**~55 (53.683)**	IncX3	
***K. oxytoca***							
Klox-45574▴	07/2016 (B2)	Rectal swab(colonization)	ST2	NDM-5	*~45 (46.161)*	IncX3	
***K. pneumoniae***							
Kpn-35963	09/2016 (A2)	Urine catheter(infection)	ST14	NDM-1, SHV-12, CTX-M-15, OXA-1	*~150 (161.324)*	IncFIB	*aacA4, dfrA14, mph*(A)
***Raoultella ornithinolytica***							
Ror-30818	09/2016 (C)	Rectal swab(colonization)	NA	NDM-1, SHV-12, CTX-M-15, OXA-1, TEM-1	*~55 (53.051)*	IncX3	

*NA, not applicable*.

a *White circles, black squares, and black triangles each indicate the NDM-like-producing isolates recovered from the same patient*.

b *Data for plasmids found in transconjugants are shown in bold; data for plasmids observed in transformants are underlined*.

NDM-like producers were collected from five Czech hospitals located in three different Czech cities. In hospital B1, an outbreak that included ten patients diagnosed with NDM-like-producing *Enterobacteriaceae* lasted the studied period. Additionally, two patients colonized or infected with NDM-like producers were reported in hospital B2. The three remaining cases were identified in three different hospitals. None of the patients, treated in hospital B1, had recently traveled abroad or had been previously hospitalized. The patient treated in hospital C was directly repatriated from a hospital in China, while clinical data weren't available for the remaining patients.

Additionally, the NDM-4-producing isolate of *E. cloacae* complex identified in 2012 (Papagiannitsis et al., 2013b), was studied.

All 19 NDM-like producers exhibited resistance to piperacillin, piperacillin-tazobactam, cephalosporins, and ertapenem (Table S2), while the observed variations in the MICs of aztreonam might reflect the presence of additional resistance mechanisms in some of the isolates. Seventeen of the NDM-like producers also exhibited resistance to ciprofloxacin; 15 were resistant to gentamicin, 13 were resistant to trimethoprim-sulfamethoxazole, 1 was resistant to amikacin, and 1 was resistant to colistin, whereas all isolates were susceptible to tigecycline.

The population structure of NDM-like-producing isolates studied by MLST is shown in Table 1. All isolates of *E. cloacae* complex, except the *E. asburiae* strain, which were recovered from hospital B1, belonged to ST182. Of note was that the NDM-4-producing isolate of *E. cloacae* complex that was isolated, in 2012, from the patient previously hospitalized in Sri Lanka (Papagiannitsis et al., 2013b) was also assigned to ST182. Two of *E. coli*, both of which were from hospital B2, belonged to ST167. *E. coli* ST167 was recently found among NDM-5-producing isolates from different healthcare institutions in China (Yang et al., 2014; Zhang et al., 2016). The two remaining *E. coli* isolates were not clonally related and belonged to different STs (ST58 and ST69). The *K. pneumoniae* isolate was assigned to the high risk clone ST14 (Woodford et al., 2011), while the *K. oxytoca* isolate was classified into ST2 that belongs to a growing international clonal complex (CC2) (Izdebski et al., 2015).

Sequencing of the PCR products revealed three *bla*_NDM_-type genes encoding the NDM-1, NDM-4, and NDM-5 enzymes (Table 1; Yong et al., 2009; Hornsey et al., 2011; Nordmann et al., 2012). NDM-5 is an NDM-1-related MβL variant that differs from NDM-1 by two amino-acid substitutions, Val88Leu and Met154Leu, the former one being its only change with NDM-4. Thirteen of the isolates, all of which were from hospital B1, were found to produce the NDM-4 MβL (Table 1). The three isolates from hospital B2 produced the NDM-5 enzyme, while the three remaining isolates that were recovered from sporadic cases in three different hospitals expressed NDM-1 carbapenemase. Additionally, most of *bla*_NDM_-like-positive isolates were confirmed to coproduce the extended-spectrum β-lactamase CTX-M-15 (*n* = 18) either alone or along with TEM-1 (*n* = 13) and/or OXA-1 (*n* = 13), whereas the *K. pneumoniae* and *R. ornithinolytica* isolates also expressed the SHV-12 enzyme. The ST58 NDM-1-producing *E. coli* isolate coproduced CMY-16, CTX-M-15, OXA-10, and TEM-1 β-lactamases.

### *bla*_NDM_-like-carrying plasmids

The *bla*_NDM_-like genes from all clinical strains were transferred by conjugation (*n* = 14) or transformation (*n* = 5) (Table 1). All *bla*_NDM_-like-positive recombinants exhibited resistance to piperacillin, piperacillin-tazobactam, cephalosporins, and ertapenem, while they remained susceptible to meropenem (Table S2). The three NDM-1-producing recombinants also exhibited resistance to aztreonam. Additionally, most of *bla*_NDM_-like-positive recombinants (*n* = 18) were susceptible to non-β-lactam antibiotics.

Plasmid analysis of NDM-4-producing donor and transconjugant strains revealed the transfer of plasmids, all of which were ~55 kb in size (Table 1). The three NDM-5-producing transformants harbored plasmids of ~45 kb, whereas the three remaining recombinants carried *bla*_NDM−1_-positive plasmids of different sizes (~55, ~150, and ~300 kb). Replicon typing showed seventeen of the plasmids, including those sizing ~45 and ~55 kb, were positive for the IncX3 allele. The *bla*_NDM−1_-positive plasmid of ~300 kb was positive for replicons R and A/C, whereas the one remaining *bla*_NDM−1_-carrying plasmid was non-typeable by the PBRT method (Carattoli et al., 2005; Johnson et al., 2012).

### Structure of *bla*_NDM_-like-carrying plasmids

**Figure 1:**
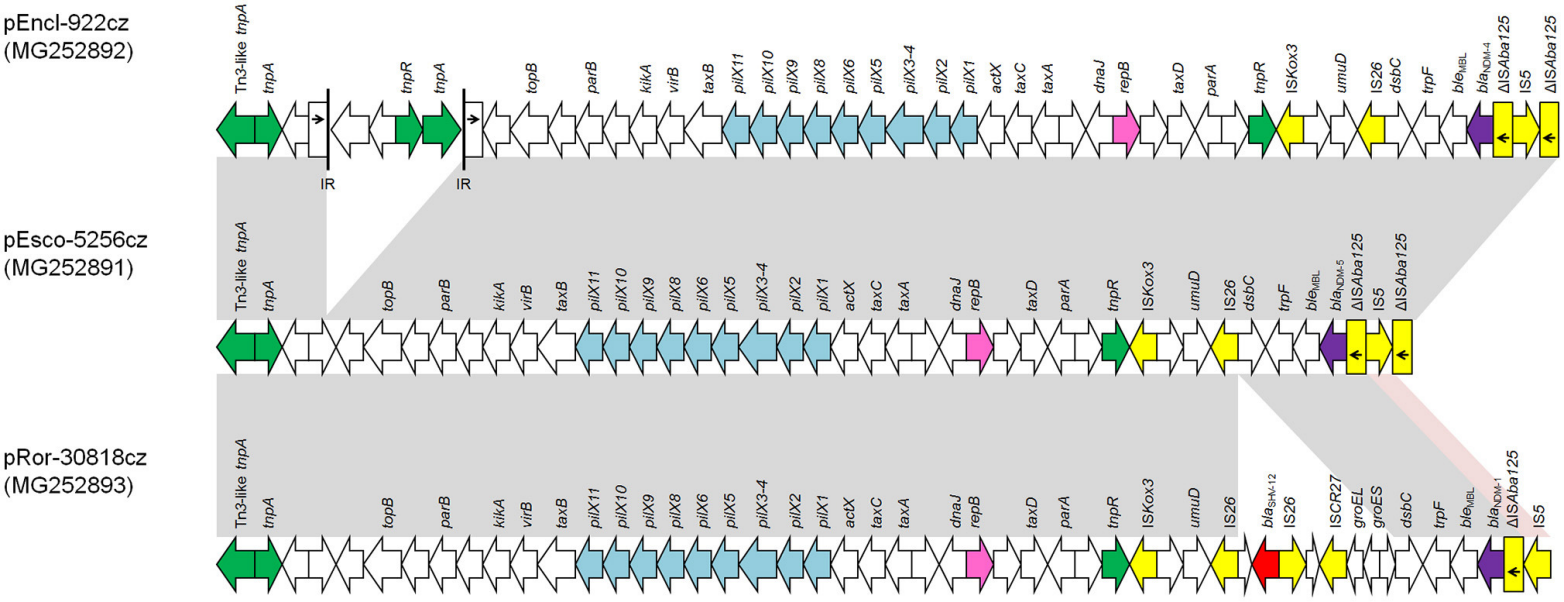
Comparison of linear maps of the NDM-like-encoding IncX3 plasmids pEncl-922cz, pEsco-5256cz, and pRor-30818cz. Arrows show the direction of transcription of open reading frames (ORFs), while truncated ORFs appear as rectangles (arrows within rectangles indicate the direction of transcription). Replicons of the plasmids are shown in pink. *bla*_NDM_-like genes are shaded purple, while other resistance genes are shown in red. IS elements and transposases are shown in yellow and green, respectively. Light blue arrows indicate genes responsible for the conjugative transfer of the plasmids. The remaining genes, including plasmid scaffold regions, are shown in white. Homologous segments (representing ≥99% sequence identity) are indicated by light gray shading, while pink shading shows inverted homologous segments.

The complete sequence of *bla*_NDM_-like-carrying plasmids representative of different plasmid sizes, replicons, and resistance genes (*n* = 10) was determined (Table 1). Sequence analysis revealed that all IncX3 *bla*_NDM_-like-carrying plasmids exhibited a high similarity to each other and to previously described NDM-like-encoding plasmids, belonging to IncX3 group, reported from worldwide (Krishnaraju et al., 2015; Zhu et al., 2016; Pál et al., 2017). The *bla*_NDM−5_-positive plasmids, pEsco-5256cz and pKlox-45574cz, were almost identical to NDM-5-encoding plasmid pNDM-QD28 (100% coverage, 99% identity) (GenBank accession no. KU167608) that was characterized from a ST167 *E. coli* in China (Zhu et al., 2016). Differences among these plasmids consisted in few SNPs (*n* = 5), almost all located in mobile elements. Similar to pNDM-QD28, no other resistance genes were detected in these plasmids. Compared to other IncX3 NDM-encoding plasmids, all *bla*_NDM−4_-encoding plasmids differed by the insertion of a Tn*3*-like transposon (nt 7108-14624 in pEncl-44578cz) downstream *topB* gene (Figure 1). The Tn*3*-like sequence was composed by the 38-bp inverted repeats (IR) of the transposon, *tnpA, tnpR*, and two ORFs encoding hypothetical proteins. Target site duplications of 5 bp (GTACC) at the boundaries of the Tn*3*-like element indicated insertion by transposition. Of note was that the sequence of pEncl-922cz, isolated in 2012 (Papagiannitsis et al., 2013b), was identical to the respective sequences of NDM-4-encoding plasmids recovered in the same hospital, during 2016. PCR screening confirmed the presence of the Tn*3*-like transposon in all NDM-4-encoding IncX3 plasmids, isolated in hospital B1, while Tn*3*-like wasn't detected in the remaining *bla*_NDM_-like-positive plasmids that belonged to IncX3 group. Furthermore, the *bla*_NDM−1_-positive plasmid, pRor-30818cz, harbored an additional 7875-bp sequence (nt 40617-48491 in pRor-30818cz) encoding the extended-spectrum β-lactamase SHV-12 (Figure 1). A similar SHV-12-encoding region was found in the IncX3 *bla*_NDM−1_-positive plasmid pKP04NDM (100% coverage, 99% identity) (GenBank accession no. KU314941) described from a *K. pneumoniae* isolate in China.

**Figure 2:**
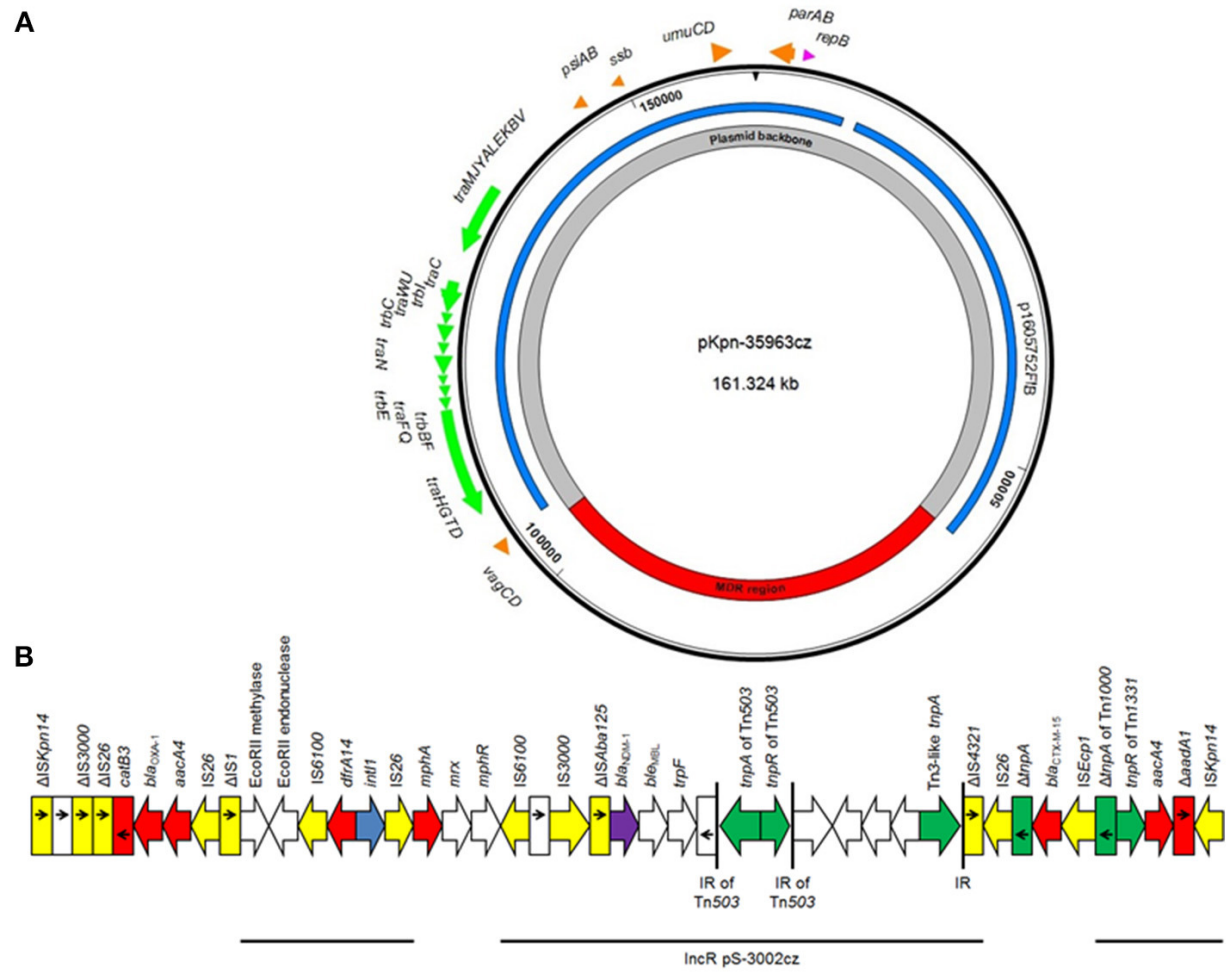
**(A)** Overview of the plasmid pKpn-35963cz. The innermost circles show the main regions of the plasmids. Similarities with other plasmids are shown in the next circle; each color represents a unique plasmid. In the outer circle, indicative genes and the direction of transcription are shown by arrows. Replicons of the plasmid are indicated as pink arrows. Genes responsible for plasmid transfer and maintenance are shown in green and orange, respectively. **(B)** Linear map of the multidrug resistance region (MDR) of the plasmid pKpn-35963cz. Arrows show the direction of transcription of open reading frames (ORFs), while truncated ORFs appear as rectangles (arrows within rectangles indicate the direction of transcription). *bla*_NDM_-like genes are shaded purple, while other resistance genes are shown in red. IS elements and transposases are shown in yellow and green, respectively. *intI1* genes are shaded blue. The remaining genes are shown in white. Thin lines below the map correspond to highly similar sequences from other plasmids.

The NDM-1-encoding plasmid pKpn-35963cz that was nontypeable by the PBRT method (Carattoli et al., 2005) was 161,324 bp in size. Plasmid pKpn-35963cz was composed of two distinct parts: a contiguous plasmid backbone of 115,998 bp (nt 1–58,655 and 103,982–161,324) and an acquired sequence of 45,326 bp (nt 58,656–103,981). The plasmid backbone, which shared similarities with the respective regions of plasmid p1605752FIB (GenBank accession no. CP022125) recovered from a pan-resistant isolate of *K. pneumoniae* from the United States, harbored regions responsible for replication [*repB* gene; IncFIB(K) replicon], conjugative transfer (*tra* and *trb* genes) and plasmid maintenance (*vagCD, psiAB, umuCD* and *parAB* operons, and *ssb* gene; Figure 2). The acquired sequence of pKpn-35963cz contained a 17,836-bp segment (nt 77,360–95,195) encoding NDM-1, which was similar to the mosaic region of pS-3002cz (99% identity) (Studentova et al., 2015). The acquired sequence of pKpn-35963cz contained two additional segments that have also been described in pS-3002cz. The first segment (nt 65,518–72,935) included genes encoding an EcoRII methylase and EcoRII endonuclease, and the class 1 integron In191 carrying the *dfrA14* resistance gene. The second segment (nt 101,342–103,981) contained fragments of transposons Tn*1000* (ΔTn*1000*) and Tn*1331* (ΔTn*1331*). ΔTn*1331* comprised *tnpR* and *aacA4* resistance gene. Furthermore the acquired sequence of pKpn-35963cz carried a macrolide resistance operon [*mph*(A)], and regions encoding OXA-1 and CTX-M-15 β-lactamases (Figure 2). In the acquired sequence of pKpn-35963cz, intact and truncated copies of several mobile elements that may have been implicated in the formation of this region were found.

**Figure 3:**
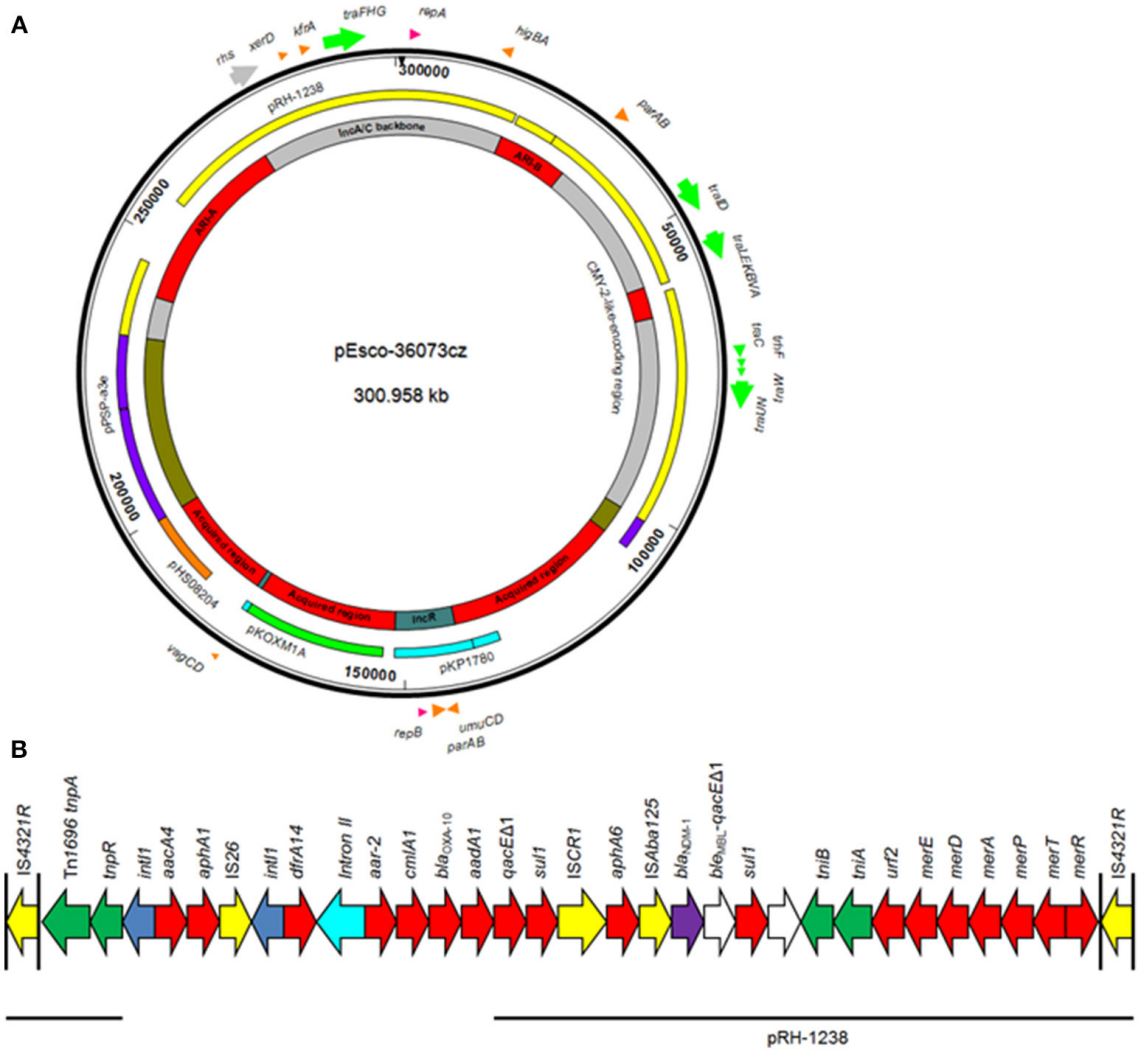
**(A)** Overview of the plasmid pEsco-36073cz. The innermost circles show the main regions of the plasmids. Similarities with other plasmids are shown in the next circle; each color represents a unique plasmid. In the outer circle, indicative genes and the direction of transcription are shown by arrows. Replicons of the plasmid are indicated as pink arrows. Genes responsible for plasmid transfer and maintenance are shown in green and orange, respectively. **(B)** Linear map of the ARI-A resistance island of the plasmid pEsco-36073cz. Arrows show the direction of transcription of open reading frames (ORFs), while truncated ORFs appear as rectangles (arrows within rectangles indicate the direction of transcription). *bla*_NDM_-like genes are shaded purple, while other resistance genes are shown in red. IS elements and transposases are shown in yellow and green, respectively. *intI1* genes are shaded blue; teal blue arrow indicates the group II intron. The remaining genes are shown in white. Thin lines below the map correspond to highly similar sequences from other plasmids.

The plasmid pEsco-36073cz, which encoded the NDM-1 carbapenemase, is 300,958 bp in size. The plasmid showed a complex structure, being composed of sequences of diverse origin (Figure 3). A 170,314-bp sequence (nt 232,204–300,958 and 1–101,559) resembled the type 1 A/C_2_ plasmid pRH-1238 (94% coverage, 99% identity; Figure 3), characterized from a *Salmonella enterica* serovar Corvallis strain isolated from a migratory wild bird in Germany (Villa et al., 2015). Analysis of A/C_2_-associated sequence by the core gene PMLST (cgPMLST) scheme (Hancock et al., 2017) indicated that it belonged to cgST3.4. The A/C_2_ backbone was composed of regions responsible for replication (*repA* gene), conjugative transfer (Tra1 and Tra2 regions), and plasmid maintenance (*higBA* and *parAB* operons and *xerD*- and *kfrA*-like genes). Apart from the backbone, pEsco-36073cz carried the *bla*_CMY−2_-like-containing region, and the ARI-B and ARI-A resistance islands, as previously described in other type 1 A/C_2_ MDR plasmids (Harmer and Hall, 2015; Papagiannitsis et al., 2016). The *bla*_NDM−1_ gene was located within ARI-A, in a genetic environment similar to those previously identified in pRH-1238 (Villa et al., 2015). However, unlike in pRH-1238, the ARI-A of pEsco-36073cz lacked the macrolide resistance determinant *mphA*-*mel*-*repAciN*. Furthermore, a class 1 integron with *aacA4* and *aphA1* gene cassettes was located between *resI* and *resII* sites of the Tn*1696* module. The ARI-A of pEsco-36073cz also carried a new integron, In1459, whose variable region comprised the *dfrA14, arr-2, cmlA1, bla*_OXA−10_, *aadA1* cassettes. Additionally, pEsco-36073cz included fragments resembling the backbone of the recently described IncR plasmid pKP1780 (Papagiannitsis et al., 2013a), and sequences previously found in the plasmid pPSP-a3e (Conlan et al., 2014) and in the chromosomes of several Gram-negative rods. Genes encoding for resistance to arsenate, cooper and mercury were identified in the three remaining acquired regions of pEsco-36073cz.

### Comparative analysis of *Enterobacter* isolates

“*In silico*” *hsp60* typing of the genome sequences (Hoffmann and Roggenkamp, 2003) showed that both isolates belonged to the recently recognized *E. xiangfangensis* species (Gu et al., 2014).

Since all isolates of *E. cloacae* complex, except the *E. asburiae* isolate, belonged to the same ST and carried the same IncX3 *bla*_NDM−4_-carrying plasmid, the WGS data of clinical strains Encl-922 and Encl-44578 were compared, using QUAST and VarScan tools, in order to examine the phylogenetic relationship of the isolates recovered in 2012 and 2016.

Comparative analysis of clinical isolates revealed that the genome of Encl-922 exhibited extensive similarity (99.87% identity) to the genome of Encl-44578. Sixteen SNPs were identified in the genome of Encl-922, compared to that of Encl-44578, five of which were located within prophage regions (Table 2). Interestingly, Encl-922 harbored three large insertions of 8,933 bp (nt 439,392–448,324 in node 2), of 17,903 bp (nt 17,786–35,688 in node 32) and of 13,165 bp (nt 1–13,165 in node 27; prophage sequence PHAGE_Salmon_SPN3UB_NC_019545). Additionally, Encl-922 harbored an insertion of 33-bp sequence (AACCCTCTCCCCAAAGGGGAGAGGGGACGATTA) located in an intergenic region. Moreover, Encl-922 showed a single nucleotide (G) deletion leading to CDS annotation change of general stress protein 39 to putative oxidoreductase YghA. Analysis of whole genome sequencing (WGS) data by PHAST web server found five intact prophage sequences (PHAGE_Haemop_HP2_NC_003315, PHAGE_Salmon_SPN3UB_NC_019545, PHAGE_Entero_mEp390_NC_019721, PHAGE_Pseudo_PPpW_3_NC_023006, and PHAGE_Salmon_SP_004_NC_021774) and one questionable prophage region (PHAGE_Entero_SfI_NC_027339), in both *E. xiangfangensis* isolates. However, Encl-922 included one additional incomplete prophage region (PHAGE_Salmon_SPN3UB_NC_019545), which was absent from the Encl-44578 genome.

**Table 2:** Summary table of 16 SNPs found between the genomes of *E. xiangfangensis* isolates Encl-44578 (reference) and Encl-922 (query).

**PROKKA name**	**Conserved domain classification**	**Enzyme commision number**	**Contig**	**SNP**	**Gene length (aa)**	**aa substitution**
–^a^	–	–	2	T64623G	–	–
–^a^	–	–	7	T88097G	–	–
Methyl viologen resistance protein SmvA	MFS transporter	–	8	T51220C	496	M293T
D-amino acid dehydrogenase small subunit	D-amino acid dehydrogenase	1.4.99.1	23	A46893G	432	S395S
NADP-dependent malic enzyme	NADP-dependent malic enzyme	1.1.1.40	2	A296564G	759	N584N
Glyoxylate/hydroxypyruvate reductase A	Glyoxylate/hydroxypyruvate reductase A	1.1.1.79	4	G113111A	312	R267H
Ribonuclease E	Ribonuclease E	3.1.26.12	4	T156395C	1035	H685R
Hypothetical protein	–	–	38	C784A	369	T239N
Hypothetical protein	Similar to protein YjaG	–	39	A24170G	196	I61V
Low-affinity gluconate transporter	Low-affinity gluconate transporter	–	6	T100479C	421	S277P
Arabinose operon regulatory protein	DNA-binding transcriptional regulator	–	12	A66284G	281	N193S
Anaerobic dimethyl sulfoxide reductase chain B	DMSO_dmsB family protein	–	35	T1976G	205	K120Q
Tail length tape measure protein	COG5281 and Phage_HK97_TLTM domain-containing protein	–	5	G24809A	1154	L824L
Tail length tape measure protein	COG5281 and Phage_HK97_TLTM domain-containing protein	–	5	T24845C	1154	A836A
Tail length tape measure protein	COG5281 and Phage_HK97_TLTM domain-containing protein	–	5	C24893A	1154	G852G
Terminase-like family protein	P family protein	–	26	G7615T	589	R485L

a *The first two SNPs are located in intergenic regions*.

Screening by PCR and sequencing identified that all *E. xiangfangensis* isolates, recovered during 2016, didn't harbor any of the four mentioned insertions. Thus, this finding indicated that *Enterobacter* isolates from 2016 differed from Encl-922.

## Discussion

The present study investigated sporadic cases and an outbreak of NDM-like-producing *Enterobacteriaceae* recovered from Czech hospitals, during 2016. Specifically, 12 NDM-4-producing isolates, which belonged to *E. xiangfangensis* (*n* = 9), *E. asburiae* (*n* = 1), *K. intermedius* (*n* = 1), and *E. coli* (*n* = 1) species, 3 NDM-5 producers of *E. coli* (*n* = 2) and *K. oxytoca* (*n* = 1) species, and one *E. coli*, one *K. pneumoniae* and one *R. ornithinolytica* producing NDM-1 MβL were characterized.

The setting that was most affected was hospital B1, in which an outbreak of NDM-4-producing ST182 *E. xiangfangensis* isolates took place. ST182 isolates of *E. cloacae* complex were previously identified in Mexico and were associated with the production of NDM-1 enzyme (Torres-González et al., 2015; Bocanegra-Ibarias et al., 2017). Of note was that the isolate of *E. cloacae* complex, isolated in 2012 from a patient who had been previously hospitalized in Sri Lanka (Papagiannitsis et al., 2013b), also belonged to ST182 and harbored an IncX3 *bla*_NDM−4_-positive plasmid being identical to respective plasmids characterized from isolates recovered from patients treated in hospital B1 (Table 1), during 2016. However, comparative genome analysis revealed the presence of four insertions in the genome of *E. xiangfangensis* Encl-922 isolate. These insertions were not found in the genomic DNA of *E. xiangfangensis* isolates from 2016, suggesting a second insertion event of NDM-4-producing *E. xiangfangensis* isolates in Czech hospitals.

In three of the patients, two different NDM-like producers were identified during their hospitalization, supposing the *in vivo* horizontal transfer of *bla*_NDM_-like-carrying plasmids. Sequencing and PCR screening data revealed the presence of the same *bla*_NDM−4_- or *bla*_NDM−5_-carrying plasmid in these isolates (Table 1). These results confirmed the hypothesis of the *in vivo* horizontal transfer of *bla*_NDM_-like-carrying plasmids.

Results from Illumina sequencing showed that IncX3 plasmids have played a major role in the dissemination of *bla*_NDM_-like genes in Czech hospitals, which is in agreement with the findings from previous studies from worldwide (Krishnaraju et al., 2015; Zhu et al., 2016; Pál et al., 2017). In the current study, three *bla*_NDM_-type genes, encoding the NDM-1, NDM-4, and NDM-5 enzymes, were associated with IncX3 plasmids exhibiting high similarity to each other. Considering also the fact that NDM-1, NDM-4, and NDM-5 differ by one or two amino-acid substitutions may indicate the possibility that *bla*_NDM_-like genes encoding NDM-1-related variants have evolved in the same plasmid type. Additionally, Illumina data showed the presence of a unique sequence, a Tn*3*-like transposon, in sequenced *bla*_NDM−4_-carrying plasmids. PCR confirmed the presence of the Tn*3*-like sequence in all transconjugants, carrying *bla*_NDM−4_-positive plasmids. Thus, the PCR targeting of the Tn*3*-like sequence was able to distinguish *bla*_NDM−4_-positive plasmids from other IncX3 plasmids carrying *bla*_NDM−1_ or *bla*_NDM−5_. On the other hand, two of the sporadic isolates carried novel NDM-1-encoding plasmids. Plasmid pKpn-35963cz that was an IncFIB(K) molecule contained an acquired sequence, encoding NDM-1 MβL, which exhibited high similarity to the mosaic region of pS-3002cz. pS-3002cs was characterized from an ST11 *K. pneumoniae* isolate, producing NDM-1 carbapenemase, identified in Czechia (Studentova et al., 2015). Whereas plasmid pEsco-36073cz was a multireplicon A/C_2_+R NDM-1-encoding plasmid, being a fusion derivative of sequences of diverse origin. Similar to other type 1 A/C_2_ plasmids (Harmer and Hall, 2015; Villa et al., 2015), the *bla*_NDM−1_ gene was located within the ARI-A resistance island.

In conclusion, the data presented here contribute to the current knowledge of NDM-like-producing *Enterobacteriaceae*. In agreement with previous studies, our findings punctuate that NDM-like producers constitute an important public threat, mainly due to the rapid horizontal transfer of IncX3 *bla*_NDM_-carrying plasmids but, also, due to further evolvement of NDM-like-encoding MDR plasmids via reshuffling.

## Author contributions

CP and JH played an important role in interpreting the results and in writing the manuscript. VJ, TB, and HZ helped to acquired data. VP, MM, AS, KC, and IB carried out experimental work. CP supervised the experiments and revised the final manuscript, which was approved by all authors.

### Conflict of interest statement

The authors declare that the research was conducted in the absence of any commercial or financial relationships that could be construed as a potential conflict of interest.
