# Supplementary material for: Characterization of NDM-Encoding Plasmids From Enterobacteriaceae Recovered From Czech Hospitals
Source: Front Microbiol. 2018 Jul 10;9:1549. doi: 10.3389/fmicb.2018.01549 (PMC6048247; doi:10.3389/fmicb.2018.01549)
Supplement: Supplementary file 1 [file Table_1.DOCX]

Table S1. Oligonucleotide primers.

| **Name** | **Sequence (5΄- 3΄)** | **Usage** | **Reference** |
| --- | --- | --- | --- |
| pNDM-F | CGTGGCTCTTGTCATGCTGA | Tn*3*-like segment mapping with pNDM4-R | This study |
| pNDM4-R | TAACGACAAAGATCAGGAGCA | Tn*3*-like segment mapping with pNDM-F | This study |
| pNDM4-F | TGCAGGTTCGCTGAAGCTG | Tn3-like segment mapping with pNDM-R | This study |
| pNDM-R | AGGGAAGTAGTCTCTGATATCT | Tn3-like segment mapping with pNDM4-F | This study |
| 922.9k-F | GATCGGCAGTAGAGGTGGA | 9-kb segment mapping with 922.9k-R | This study |
| 922.9k-R | TACCAAAGCAACAGCTGACG | 9-kb segment mapping with 922.9k-F | This study |
| 922.13k-F | GCTCTACAGCAGCGTTCCAG | 13-kb segment mapping with 922.13k-R | This study |
| 922.13k-R | AGTGGGAAAGCGTTGCAGATC | 13-kb segment mapping with 922.13k-F | This study |
| 922.18k-F | TGATACATGAACAAGGCAGATG | 18-kb segment mapping with 922.18k-R | This study |
| 922.18k-R | GTGCGATCAATGGTTAACTCA | 18-kb segment mapping with 922.18k-F | This study |
| 922.33b-F | ATGGCGAAACTGCCCTCGA | 33-bp insertion mapping with 922.33b-R | This study |
| 922.33b-R | TCTGTCTGAACGTGCTGGCT | 33-bp insertion mapping with 922.33b-R | This study |
