# Supplementary material for: Characterization of NDM-Encoding Plasmids From Enterobacteriaceae Recovered From Czech Hospitals
Source: Front Microbiol. 2018 Jul 10;9:1549. doi: 10.3389/fmicb.2018.01549 (PMC6048247; doi:10.3389/fmicb.2018.01549)
Supplement: Supplementary file 2 [file Table_2.DOCX]

Table S2. Antimicrobial susceptibility of NDM-like-producing clinical and recombinant strains.

| **Isolate** | **MIC (mg/l) of:^a^** | | | | | | | | | | | | | | | |
| --- | --- | --- | --- | --- | --- | --- | --- | --- | --- | --- | --- | --- | --- | --- | --- | --- |
|  | **Pip** | **Tzp** | **Ctx** | **Caz** | **Fep** | **Atm** | **Mem** | **Etp** | **Gen** | **Amk** | **Cmp** | **Tet** | **Sxt** | **Cip** | **Col** | **Tgc** |
| Encl-922 | >128 | >128 | >8 | >16 | >16 | >16 | 4 | >2 | 16 | 2 | 16 | >32 | >4 | >8 | 0.25 | 1 |
| Trc Encl-922 | 128 | 128 | >8 | >16 | 16 | ≤0.12 | 1 | 2 | ≤0.5 | ≤0.25 | 4 | 0.5 | ≤0.03 | ≤0.06 | 0.25 | 0.25 |
| Encl-66918 | >128 | >128 | >8 | >16 | >16 | >16 | 4 | >2 | 16 | 2 | 16 | >32 | >4 | >8 | 0.25 | 1 |
| Trc Encl-66918 | 128 | 128 | >8 | >16 | 8 | ≤0.12 | 1 | 2 | ≤0.5 | ≤0.25 | 4 | 1 | ≤0.03 | ≤0.06 | 0.25 | 0.25 |
| Encl-89040 | >128 | >128 | >8 | >16 | >16 | >16 | 2 | >2 | 16 | 4 | 16 | >32 | >4 | >8 | 0.25 | 1 |
| Trc Encl-89040 | 128 | 128 | >8 | >16 | 8 | ≤0.12 | 1 | 2 | ≤0.5 | ≤0.25 | 4 | 1 | ≤0.03 | ≤0.06 | 0.25 | 0.25 |
| Encl-44578 | >128 | >128 | >8 | >16 | >16 | >16 | 2 | >2 | 16 | 1 | 16 | >32 | >4 | >8 | 0.25 | 1 |
| Trc Encl-44578 | 128 | 128 | >8 | >16 | 8 | ≤0.12 | 1 | 2 | ≤0.5 | ≤0.25 | 4 | 1 | ≤0.03 | ≤0.06 | 0.25 | 0.25 |
| Encl-89485 | >128 | >128 | >8 | >16 | >16 | >16 | 4 | >2 | 16 | 1 | 16 | >32 | >4 | >8 | 0.25 | 1 |
| Trc Encl-89485 | 128 | 128 | >8 | >16 | 8 | ≤0.12 | 1 | 2 | ≤0.5 | ≤0.25 | 4 | 1 | ≤0.03 | ≤0.06 | 0.25 | 0.25 |
| Encl-91221 | >128 | >128 | >8 | >16 | >16 | >16 | 4 | >2 | >32 | 1 | 16 | >32 | >4 | >8 | 0.25 | 1 |
| Trc Encl-91221 | 128 | 128 | >8 | >16 | 8 | ≤0.12 | 1 | 2 | ≤0.5 | ≤0.25 | 4 | 1 | ≤0.03 | ≤0.06 | 0.25 | 0.25 |
| Encl-93141 | >128 | >128 | >8 | >16 | >16 | >16 | 4 | >2 | 16 | 1 | 16 | >32 | 0.5 | >8 | ≤0.12 | 1 |
| Trc Encl-93141 | 128 | 128 | >8 | >16 | 8 | ≤0.12 | 1 | 2 | ≤0.5 | ≤0.25 | 4 | 1 | ≤0.03 | ≤0.06 | 0.25 | 0.25 |
| Encl-98042 | >128 | >128 | >8 | >16 | >16 | >16 | 2 | >2 | 32 | 1 | 16 | >32 | 1 | >8 | 0.25 | 1 |
| Trc Encl-98042 | 128 | 128 | >8 | >16 | 8 | ≤0.12 | 1 | 2 | ≤0.5 | ≤0.25 | 4 | 1 | ≤0.03 | ≤0.06 | 0.25 | 0.25 |
| Encl-98047 | >128 | >128 | >8 | >16 | >16 | >16 | 2 | >2 | 16 | 1 | 16 | >32 | >4 | >8 | 0.25 | 1 |
| Trc Encl-98047 | 128 | 128 | >8 | >16 | 8 | ≤0.12 | 1 | 2 | ≤0.5 | ≤0.25 | 4 | 1 | ≤0.03 | ≤0.06 | 0.25 | 0.25 |
| Encl-98546 | >128 | >128 | >8 | >16 | >16 | >16 | 16 | >2 | 16 | 1 | 16 | >32 | 0.25 | 0.12 | 0.25 | 1 |
| Trc Encl-98546 | 128 | 128 | >8 | >16 | 8 | ≤0.12 | 1 | 2 | ≤0.5 | ≤0.25 | 4 | 1 | ≤0.03 | ≤0.06 | 0.25 | 0.25 |
| Enas-80654 | >128 | >128 | >8 | >16 | >16 | 0.5 | 16 | >2 | ≤0.25 | ≤0.5 | 16 | 8 | 0.12 | 8 | >16 | 1 |
| Trc Enas-80654 | 128 | 128 | >8 | >16 | 8 | ≤0.12 | 1 | 2 | ≤0.5 | ≤0.25 | 4 | 1 | ≤0.03 | ≤0.06 | 0.25 | 0.25 |
| Klin-51781 | >128 | 128 | >8 | >16 | >16 | >16 | 2 | >2 | 16 | 1 | 16 | >32 | 1 | 1 | 0.25 | 1 |
| Trc Klin-51781 | 128 | 128 | >8 | >16 | 16 | ≤0.12 | 1 | 2 | ≤0.5 | ≤0.25 | 4 | 0.5 | ≤0.03 | ≤0.06 | 0.25 | 0.25 |
| Esco-36073 | >128 | >128 | >8 | >16 | >16 | 16 | 2 | >2 | 8 | 4 | >32 | >32 | >4 | ≤0.06 | 0.25 | 0.25 |
| Trc Esco-36073 | >128 | >128 | >8 | >16 | >16 | 16 | 2 | >2 | 2 | 2 | >32 | 32 | >4 | ≤0.06 | 0.25 | 0.25 |
| Esco-4382 | >128 | >128 | >8 | >16 | >16 | ≤0.12 | 1 | >2 | 32 | 1 | 4 | >32 | >4 | >8 | 0.25 | 0.25 |
| Trc Esco-4382 | 128 | 128 | >8 | >16 | 16 | ≤0.12 | 1 | 2 | ≤0.5 | ≤0.25 | 4 | 0.5 | ≤0.03 | ≤0.06 | 0.25 | 0.25 |
| Esco-5256 | >128 | >128 | >8 | >16 | >16 | 1 | 8 | >2 | ≤0.25 | ≤0.5 | 4 | >32 | >4 | >8 | ≤0.12 | 0.12 |
| Trf Esco-5256 | 128 | 128 | >8 | >16 | >16 | ≤0.12 | 2 | >2 | ≤0.5 | ≤0.25 | 2 | 0.5 | ≤0.03 | ≤0.06 | 0.25 | 0.25 |
| Esco-14290 | >128 | >128 | >8 | >16 | >16 | >16 | 2 | 2 | 16 | 1 | 16 | >32 | >4 | >8 | 0.25 | 0.12 |
| Trf Esco-14290 | 128 | 128 | >8 | >16 | >16 | ≤0.12 | 2 | >2 | ≤0.5 | ≤0.25 | 2 | 0.5 | ≤0.03 | ≤0.06 | 0.25 | 0.25 |
| Klox-45574 | >128 | >128 | >8 | >16 | >16 | >16 | 4 | >2 | ≤0.25 | ≤0.5 | >32 | >32 | >4 | 2 | ≤0.12 | 0.5 |
| Trf Klox-45574 | 128 | 128 | >8 | >16 | >16 | ≤0.12 | 2 | >2 | ≤0.5 | ≤0.25 | 2 | 0.5 | ≤0.03 | ≤0.06 | 0.25 | 0.25 |
| Kpn-35963 | >128 | >128 | >8 | >16 | >16 | >16 | 2 | >2 | 0.5 | 4 | 16 | >32 | 1 | >8 | 0.25 | 0.5 |
| Trf Kpn-35963 | >128 | 64 | >8 | >16 | 8 | >16 | 0.5 | 2 | 0.5 | 4 | 1 | 16 | 0.25 | ≤0.06 | 0.25 | 0.25 |
| Ror-30818 | >128 | >128 | >8 | >16 | >16 | >16 | 8 | >2 | >32 | >64 | >32 | 4 | >4 | >8 | 0.25 | 1 |
| Trf Ror-30818 | >128 | >128 | >8 | >16 | 16 | >16 | 1 | >2 | 0.5 | 4 | 1 | 16 | 0.25 | ≤0.06 | 0.25 | 0.25 |

^a^Pip, piperacillin; Tzp, piperacillin-tazobactam (inhibitor fixed at 4 mg/l); Ctx, cefotaxime; Caz, ceftazidime; Fep, cefepime; Atm, aztreonam; Mem, meropenem; Etp, ertapenem; Gen, gentamicin; Amk, amikacin; Cmp, chloramphenicol; Tet, tetracycline; Sxt, trimethoprim-sulfamethoxazole; Cip, ciprofloxacin; Col, colistin; Tgc, tigecycline.
